# The development of *Leishmania turanica* in sand flies and competition with *L. major*

**DOI:** 10.1186/1756-3305-5-219

**Published:** 2012-10-02

**Authors:** Alsu Chajbullinova, Jan Votypka, Jovana Sadlova, Katerina Kvapilova, Veronika Seblova, Jakub Kreisinger, Milan Jirku, Chizu Sanjoba, Sambuu Gantuya, Yoshitsugu Matsumoto, Petr Volf

**Affiliations:** 1 Department of Parasitology, Fac. Sci, Charles University in Prague, Prague, Czech Republic; 2 Department of Zoology, Fac. Sci, Charles University in Prague, Prague, Czech Republic; 3 Biology Centre, Institute of Parasitology, Ceske Budejovice, Czech Republic; 4 Department of Molecular Immunology, University of Tokyo, Tokyo, Japan

**Keywords:** *Leishmania turanica*, *L. major*, Mixed infections, Competition, Genetic exchange, Vector competence, *Phlebotomus*

## Abstract

**Background:**

In Central Asian foci of zoonotic cutaneous leishmaniases, mixed infections of *Leishmania turanica* and *L. major* have been found in a reservoir host (the great gerbil, *Rhombomys opimus*) as well as in the sand fly vector *Phlebotomus papatasi*, but hybrids between these two *Leishmania* species have never been reported. In addition, the role of sand fly species other than *P. papatasi* in *L. turanica* circulation is not clear.

**Methods:**

In this work we compared the development of *L. turanica* in three sand fly species belonging to different subgenera. In addition, we studied experimental co-infections of sand flies by both *Leishmania* species using GFP transfected *L. turanica* (MRHO/MN/08/BZ18(GFP^+^)) and RFP transfected *L. major* (WHOM/IR/-/173-DsRED(RFP^+^)). The possibility of *Leishmania* genetic exchange during the vectorial part of the life cycle was studied using flow cytometry combined with immunofluorescent microscopy.

**Results:**

Late-stage infections of *L. turanica* with frequent colonization of the stomodeal valve were observed in the specific vector *P.* (*Phlebotomus*) *papatasi* and in the permissive vector *P.* (*Adlerius*) *arabicus*. On the other hand, in *P. sergenti* (the specific vector of *L. tropica*), *L. turanica* promatigotes were present only until the defecation of bloodmeal remnants. In their natural vector *P. papatasi*, *L. turanica* and *L. major* developed similarly, and the spatiotemporal dynamics of localization in the sand fly gut was the same for both leishmania species. Fluorescence microscopy in combination with FACS analyses did not detect any *L. major / L. turanica* hybrids in the experimental co-infection of *P. papatasi* and *P. duboscqi*.

**Conclusion:**

Our data provide new insight into the development of different leishmania parasite species during a mixed infection in the sand fly gut. Despite the fact that both *Leishmania* species developed well in *P. papatasi* and *P. duboscqi* and did not outcompete each other, no genetic exchange was found. However, the ability of *L. turanica* to establish late-stage infections in these specific vectors of *L. major* suggests that the lipophosphoglycan of this species must be identical or similar to that of *L. major*.

## Background

Leishmaniases are vector-born diseases transmitted by phlebotomine sand flies and caused by a protozoan flagellate of the genus *Leishmania*. *Leishmania major*, the main causative agent of zoonotic cutaneous leishmaniasis (ZCL) is spread over a wide area in Africa, the Middle East and Asia, while *L. turanica*, a species less pathogenic to humans [1] is found mainly in Central Asia (reviewed by [2]).

In Central Asia, the great gerbil (*Rhombomys opimus*) is well documented as a reservoir host for both *L. turanica* and *L. major*, which may also occur in sympatry with nonpathogenic *L. gerbilli* (reviewed by [3]). All three *Leishmania* species have been found to coexist in a single gerbil, and mixed infections of *L. turanica* and *L. major* are frequent [4-7]. In sympatric areas, mixed infections of *L. major* and *L. turanica* have a significant effect on the circulation of *L. major* in the wild; co-infection with *L. turanica* results in an increased duration of *L. major* infection in gerbil populations and enables the persistence of *L. major* to the subsequent transmission season [5,8]. *Phlebotomus papatasi* is believed to be the vector for both *Leishmania* species [3], and mutual circulation in *P. papatasi* has been recently reported using molecular methods [9,10].

A different situation regarding the transmission cycle has been described in China and Mongolia. In these areas, the presence of *L. major* and its main vector *P. papatasi* has not been demonstrated, and only the circulation of *L. turanica* and *L. gerbilli* in *R. opimus* has been found [1,4,11]. In addition to *P.* (*Phlebotomus*) *papatasi*, *L. turanica* has been reported from two members of the subgenus *P. andrejevi* and *P. mongolensis* in China [1]. However, the role of these vectors in the circulation of *L. turanica* is not clear.

In the present study, we compared the development of *L. turanica* in three sand fly species distributed in Central Asia and the Middle East: *P. papatasi* and *P. (Paraphlebotomus) sergenti* represent vectors specific for *L. major* and *L. tropica* (reviewed by [12]), respectively, while *Phlebotomus* (*Adlerius*) *arabicus* was chosen as a member of the permissive vectors susceptible to various *Leishmania* spp. (reviewed by [13]). Finding that *L. turanica* and *L. major* develop similarly and co-localize in *P. papatasi* midgut led us to attempt to detect possible genetic hybrids using fluorescent marked *L. turanica* and *L. major* strains. Genetic exchange in *Leishmania* is a very rare event (reviewed by [14]) but has important epidemiological consequences; it can rapidly enhance the fitness of the parasite [15,16] and the transmission of hybrid offspring can utilize new vectors in their circulation [17,18].

## Methods

### *Leishmania* parasites and sand fly colonies

*Leishmania* transfection by GFP and RFP plasmids was adopted from our previous work [19] and for hybrid (*L. major* / *L. turanica*) visualization we applied the method previously used for detection of *L. donovani* hybrids in sand flies co-infected with a mixture of two parental GFP/RFP expressing strains [20]. These hybrid cells simultaneously emit at GFP and RFP wavelengths and showed a patched appearance of red and green fluorescence under the microscope [20]. *L. major* WHOM/IR/-/173-DsRED(RFP^+^) and *L. turanica* MRHO/MN/08/BZ18(GFP^+^) were maintained at 23°C on RPMI-1640 medium and Schneider-D medium (3:1) supplemented with 10% fetal calf serum and amicin (200 μl/ml). For the GFP-marked *L. turanica*, the selection antibiotic nourseothricin (1 μl/ml) was added to the culture medium. Cultures were subcultured every 3 to 5 days. Prior to sand fly infections, parasites were washed by centrifugation and resuspended in saline solution. Laboratory colonies of four sand fly species, *P. papatasi* (originally from Turkey), *P. arabicus* (Israel), *P. sergenti* (Israel) and *P. duboscqi* (Senegal), were maintained on 50% sucrose at 25°C as described previously [21].

### Experimental infections of sand flies

Sand fly females (5 to 8 days old) were fed through a chick skin membrane on heat-inactivated rabbit blood (Bioveta, Ivanovice) containing 4 to 5 day old L*eishmania* promastigotes. The cell density (promastigotes/ml) for all *L. turanica* single infections in three different sand fly species (114 specimens of *P. papatasi*, 128 *P. arabicus* and 78 *P. sergenti*) was 1x10^6^; the same cell density was used for single infections of 68 specimens of *P. papatasi* with *L. major*. Three different cell densities were used for mixed infections of *L. major* and *L. turanica* in 114, 119 and 61 specimens of *P. papatasi*, respectively: 1x10^5^ (combining 5x10^4^*L. major* + 5x10^4^*L. turanica*), 1x10^6^ (5x10^5^*L. major* + 5x10^5^*L. turanica*), and 1x10^7^ (5x10^6^*L. major* + 5x10^6^*L. turanica*). Blood-engorged sand fly females were separated and maintained as described above, and dissected 2, 5, 9, and 12 days post infection (DPI). The localization and intensity of infection (number of promastigotes) in the midgut were estimated under a fluorescence microscope (Olympus BX51 with an Olympus DP 70 camera). The intensity of infection was graded as being light (< 100 parasites/gut), moderate (100–1000 parasites/gut), or heavy (> 1000 parasites/gut) as described previously [22,23]. Parasite location was categorized as being in the abdominal midgut (AMG), thoracic midgut (TMG), cardia region (CAR), or colonized stomodeal valve (SV-C). Each experiment was repeated at least twice. The total number of dissected sand flies in various experiments is indicated above the bars.

In addition, we searched for putative *L. major/L. turanica* hybrids. As *L. donovani* hybrids were previously reported as early as on day 2 [20], groups of 30–50 females were dissected into physiological solution at different DPI and the midgut homogenate was filtered using a 30 μm filter (Partec) into PBS buffer. To maximize the chance of identifying hybrids, two series of experiments differing in sand fly species and flow cytometer type were carried out. Both cytometers were calibrated using positive controls (GFP^+^ and RFP^+^ transfected *Leishmania* line) and a negative control (wild type *Leishmania* strain).

In the first series of experiments, *Leishmania* from *P. papatasi* midguts were sorted by a FACS Vantage SE cell sorter with laser emitting at 488 nm wavelength and detection of emission at 530/30 nm (GFP^+^) and 585/42 nm (RFP^+^). Sorting was performed in two steps: in the first step all GFP positive events were separated; then, from this presorted material all double positive events were put into a glycerol drop containing 1% formaldehyde. Since the sorter is not designed for optimal excitation of RFP^+^ events, the total number of RFP^+^ events was determined by an LSRII flow cytometer (BD Biosciences: excitation with the 561 nm laser, emission detection at 585/15 nm).

In the second series of experiments, *P. (Phlebotomus) duboscqi*, another natural vector of *L. major*, was used. *Leishmania* from *P. duboscqi* midguts were sorted by a BD Influx cytometer equipped by (i) laser emitting at 488 nm wavelengths and detection of emission at 530/40 nm (GFP) and (ii) laser emitting at 561 nm wavelengths and detection of emission at 585/29 nm (RFP). These parameters allowed the separation of double positive events in one step (and without the additional use of the LSRII cytometer).

Sorted material from both series was immediately checked visually on a confocal microscope (Olympus FV1000) to distinguish and exclude sorted artifacts from potential hybrids expressing both green and red signals.

### Statistical analysis of experimental infections

The software STATISTICA and R 2.13.1 (R Development Core Team [24]) were used for data analyzes. In single infections, the χ2 test was used to compare infection intensities (heavy, moderate, light, zero) and localizations (AMG, TMG, CAR, SV-C).

In mixed infections, the permutation-based approach [25] was used to assess differences in the proliferation pattern between *L. turanica* and *L. major* in *P. papatasi*. In particular, the relative prevalence of both *Leishmania* species within each infected sand fly was scored using a three level scale: -1, intensity of *L. turanica* < *L. major*; 0, intensity of *L. turanica* < *L. major*, and +1, intensity of *L. turanica* > *L. major*. The null hypothesis that infection intensities do not differ between both *Leishmania* species cannot be excluded if confidence intervals of the mean of relative prevalence scores overlap with zero. Hence to obtain 95% confidence intervals of relative prevalence scores we bootstrapped them 1 000 times. In the next step, three separate permutation based tests were performed for different initial infection doses, to evaluate the consistency of results across different initial conditions.

In the next step we evaluated the spatio-temporal dynamics of each *Leishmania* species infection in the digestive tract of sand flies. Two separate ANOVA models for *L. turanica* and *L. major* data were fitted to test for the relationship between the localization of infection in a particular part of the digestive tract and days after the infective feed (DPI). We further included the interaction between the localization of infection and initial infection dose, to test for the variation caused by this factor. In the next step, we scored the relative spatial distribution of both *Leishmania* species using the following three level scale: -1, *L. turanica* present in a more distal part of the digestive tract than *L. major*; 0, both *Leishmania* species present in the same part of the digestive tract; and +1, *L. turanica* present in a more proximal part of the digestive tract than *L. major*. The null hypothesis that the mean of these scores does not differ from zero was tested using bootstrap estimates of confidence intervals as described above.

## Results

### Comparing the development of *L. turanica* in three sand fly species

**Figure 1:**
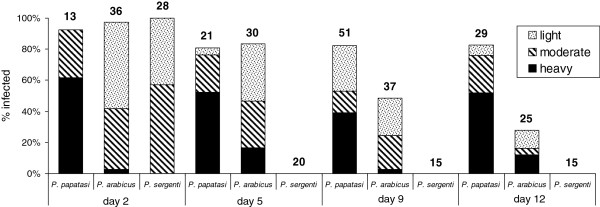
** Infection rates of *Leishmania turanica* in *Phlebotomus papatasi, P. arabicus* and *P. sergenti*.** Infection rates were examined 2 to 12 days after the infective bloodmeal. Intensity of infection is categorized as: light, less than 100; moderate, 100 to 1000 and heavy, more than 1000 parasites per gut. The number of dissected sand flies is indicated above the bars.

Infections of *L. turanica* differed significantly between all tested sand fly vector species (Figure 1): *P. papatasi* vs. *P. arabicus* (Pearson Chi-square: 50.62, df = 3, p < 0.001); *P. papatasi* vs*. P. sergenti* (Pearson Chi-square: 65.95, df = 3, p < 0.001); *P. arabicus* vs. *P. sergenti* (Pearson Chi-square: 22.52, df = 3, p < 0.001).

On day 2 PI, midgut infection rates were high (~90%) in all studied sand fly vector species and parasites were located only in the blood meal in the AMG. On day 5 PI, when the bloodmeal was digested and remnants defecated in all three species, infection rates remained high (~80%) in *P. papatasi* and *P. arabicus*, but no *Leishmania* were found in *P. sergenti*. In *P. papatasi* most infections were heavy or moderate, whereas significantly weaker infections occurred in *P. arabicus* (Pearson Chi-square: 10.58, df = 3, p = 0.01) (Figure 1).

On days 9 and 12 PI, the difference in infection rates of *P. papatasi* and *P. arabicus* became more marked (>80% in *P. papatasi* but only 48 and 28% in *P. arabicus*); in both species, however, parasites migrated anteriorly to colonize the thoracic midgut and stomodeal valve. Again, no parasites were found in *P. sergenti* (Figure 1).

### Comparing single infections of *L. turanica* and *L. major* in *P. papatasi*

**Figure 2:**
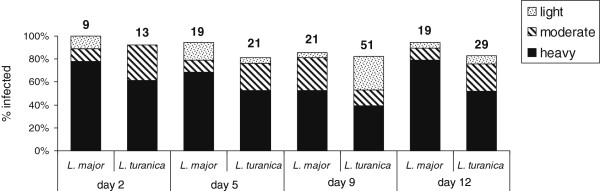
** Single infection of *Leishmania turanica* and *L. major* in *Phlebotomus papatasi*.** Infections were examined 2 to 12 days after the infective bloodmeal. Intensity of infection is categorized as: light, less than 100; moderate, 100 to 1000 and heavy, more than 1000 parasites per gut. The number of dissected sand flies is indicated above the bars.

*Leishmania turanica* and *L. major* single infections gave high infection rates (~80%) on all examined DPI (Figure 2); however, the intensity of the *L. major* infection was significantly higher than that of *L. turanica* (Pearson Chi-square: 8.81, df = 3, p = 0.03). Differences in the gut locations between the *L. turanica* and *L. major* infections were insignificant on all examined DPI (Pearson Chi-square: 0.54, df = 3, p = 0.91). On day 2 PI, all parasites were in the residual bloodmeal in the abdominal midgut. By day 5 PI, when the bloodmeal had been digested and remnants defecated, parasites had migrated anteriorly to the thoracic midgut and cardia region (*L. turanica* 64%; *L. major* 78%), but colonization of the stomodeal valve was observed in only one female infected with *L. major*. On day 9 PI, an intense infection with colonization of the stomodeal valve developed in 50% of *L. major* and 48% *L. turanica* infections; on day 12 PI, colonization of the stomodeal valve was found in 78% *L. major* and 46% *L. turanica*.

### The development of mixed infections of *L. turanica* / *L. major* in *P. papatasi*

**Figure 3:**
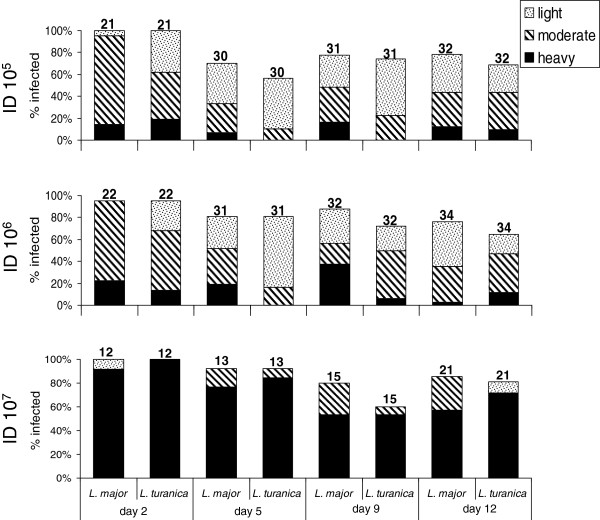
** Mixed infection of *Leishmania turanica* and *L. major* in *Phlebotomus papatasi*.** Infections were examined 2 to 12 days after the infective bloodmeal according to three different infection doses (ID). Intensity of infection is categorized as: light, less than 100; moderate, 100 to 1000 and heavy, more than 1000 parasites per gut. The number of dissected sand flies is indicated above the bars.

The intensity of *L. turanica* and *L. major* infections was similar in the majority of *P. papatasi* females (55.4% cases). *Leishmania major* overgrew *L. turanica* in 30.9% cases, while the opposite situation was observed in 13.7% of individuals. Three different doses of *L. turanica/L. major* mixed infections in *P. papatasi* were analyzed separately (see Figure 3). Bootstrap estimates of 95% CI for relative prevalence scores do not overlap with zero (Mean = −0.171, 95% CI range = −0.248 ~ −0.121, p < 0.001), suggesting that *L. major* is in general slightly more prevalent that *L. turanica*. However, the effect size of this difference was relatively low: the tendency for *L. major* infections to have higher intensities was revealed in groups that were exposed to 10^5^ and 10^6^ initial infection doses (Mean = −0.262, 95% CI range = −0.377 ~ −0.149, p < 0.001 and Mean = −0.212, 95% CI range = −0.327 ~ −0.092, p < 0.001, respectively), yet we did not find any difference between *L. turanica* and *L. major* when initial infection doses were 10^**7**^ (Mean = +0.045, 95% CI range = −0.092 ~ +0.184, p = 0.719). Significant variation of relative prevalence scores for *L. turanica* vs. *L major* according to initial infection doses was detected using the chi square test (df = 4, χ^2^ = 11.04, p = 0.026). (Figure 3).

**Figure 4:**
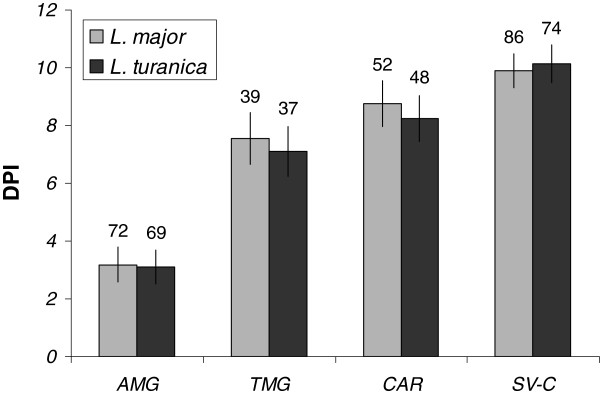
** Localization of the infections depending on days after the infective bloodmeal (DPI).** The height of light (*Leishmania major*) or dark (*L. turanica*) bars represent the average (mean +/− 95% confidence interval) DPI when *L. major*/*L. turanica* infection colonized the relevant part of the gut: abdominal midgut (AMG), thoracic midgut (TMG), cardia region (CAR) and stomodeal valve (SV-C). The numbers above the bars indicate the number of infected sand fly females with the relevant gut localization

**Figure 5:**
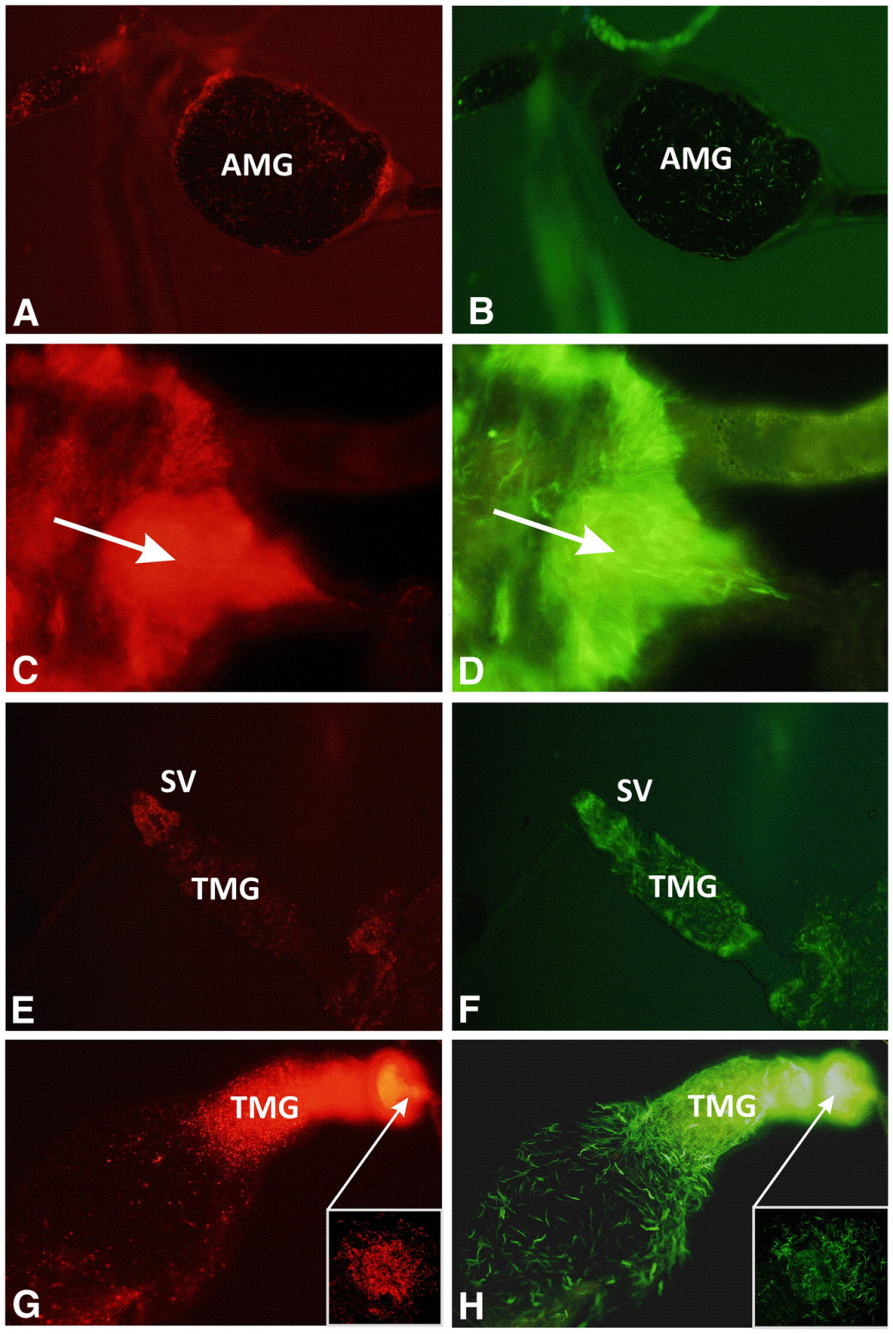
** Development and localization of mixed *Leishmania turanica* and *L. major* infections in *P. papatasi* guts. ***L. turanica* was transfected by GFP (**B**, **D**, **F**, **H**) while *L. major* was transfected by RFP (**A**, **C**, **E**, **G**). In the early stage of infection (2 DPI) parasites multiply in the bloodmeal in the abdominal midgut, AMG (**A**, **B**). After defecation (5 DPI) parasites reach the apical part of the AMG (arrow) (**C**, **D**), while colonization of the thoracic midgut (TMG) and stomodeal valve (SV) is typical for late infections (9 DPI) (**E**, **F**). On day 12 DPI, a heavy infection of the colonized SV (arrow) is prevalent (**G**, **H**).

The interaction between initial infection dose and infection localization was not significant in *L. major* (F_(6,237)_ = 1.702, p = 0.121)*,* yet this interaction was marginally significant in *L. turanica* (F_(6,216)_ = 2.17, p = 0.047). Aside from this interactive effect, both strains exhibited a strong relationship between the infection localization and DPI (One-way ANOVA: F_(3,245)_ = 100.15, p < 0.001 and F_(3,224)_ = 103.39, p < 0.001 for *L. major* and *L. turanica* respectively). This pattern was comparable for both *Leishmania* species (Figure 4), and differences between *L. turanica* and *L. major* in the time-dependent colonization of various sand fly gut sections were non-significant. *Leishmania major* and *L. turanica* were present in the same part of the digestive tract in the vast majority of dissected individuals (182 cases, 82.7%). The number of cases in which *L. major* or *L. turanica* were localized in more proximal parts of the digestive tract than the other strain were comparable (17 cases, 7.7% and 21 cases, 9.5% respectively). Bootstrap based, 95% confidence intervals for relative position scores of both strains in the digestive tract overlapped with zero (95% CI range: -0.03634 ~ +0.0545, p = 0.244). Furthermore, no deviation from the null hypothesis expectation was detected when performing this test separately for individual initial infection doses. Finally, the relative localization of the infection did not vary significantly according to initial infection doses as demonstrated using the chi square test (df = 4, χ^2^ = 1.64, p = 0.802). This indicates that there is no pronounced difference in the spatio-temporal dynamic of colonization of the digestive tract between both *Leishmania* species. The development of *L. turanica* and *L. major* mixed infections and localization in *P. papatasi* midguts is illustrated in Figure 5.

**Figure 6:**
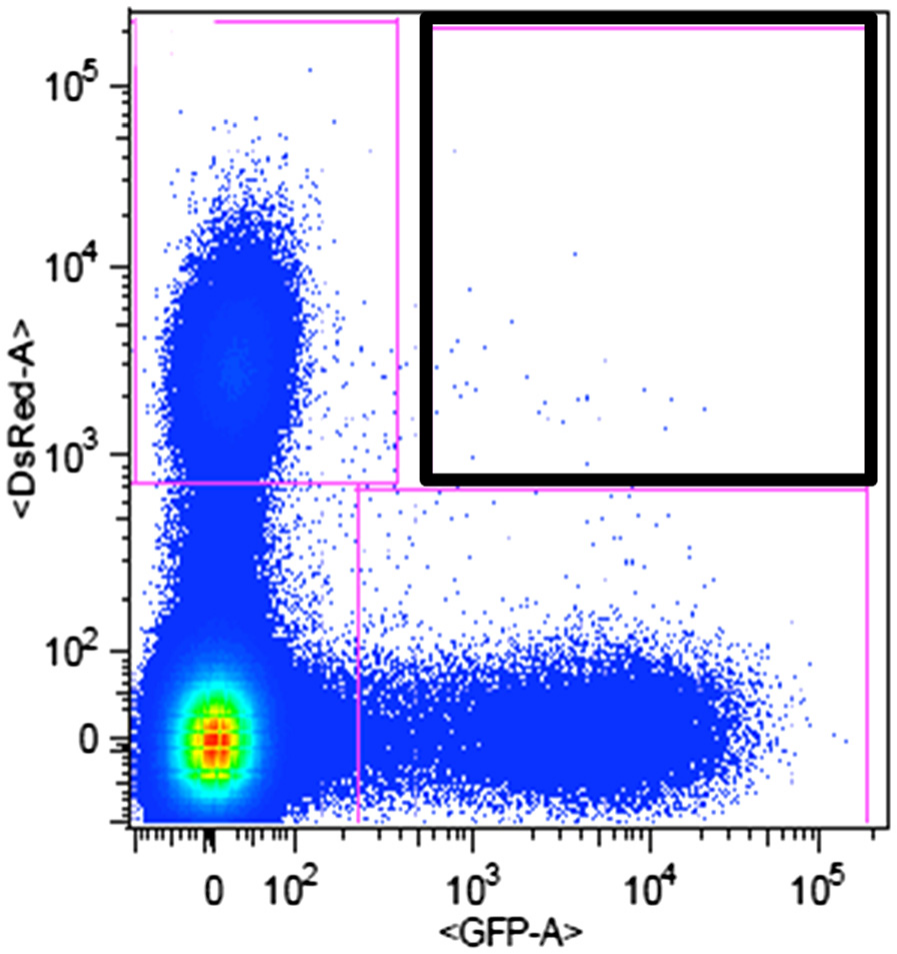
** FACS analysis.** An example of FACS analysis of the first series of experiments with *P. papatasi* (50 guts) at day 2 PI. The black rectangle shows the region sorted and screened for hybrids.

The attempt to detect *L. turanica* and *L. major* hybrids was not successful. In the first series of experiments, a total of 100 co-infected females of *P. papatasi* were dissected on 2 DPI and 80 females on 9 DPI. Putative hybrid cells were sorted by a FACS Vantage SE cell sorter and observed by a confocal fluorescence microscope. For example, 31 putative hybrids were sorted at 2 DPI (see Figure 6); however, all were found to be artifacts (sand fly gut remnants). In the second series of experiments, a total of 130 co-infected *P. duboscqi* females were dissected on days 2, 9 and 14 PI. Samples were analyzed by a BD Influx cell sorter but again no hybrids were confirmed by fluorescent microscopy.

## Discussion

This study provides experimental evidence that *L. turanica* develops late-stage infections in *P. papatasi*. According to the criteria of Killick-Kendrick [26], this finding, together with the well-known anthropophily of this sand fly species and repeated isolation of *L. turanica* from wild-caught *P. papatasi* in Central Asia (reviewed by [3]) and Iran [9,10], incriminate *P. papatasi* as the vector of *L. turanica*.

Comparing the development of *L. turanica* and *L. major* in *P. papatasi* revealed high similarity and found that statistical differences are not significant from a biological point of view. Although the differences in intensities between *L. turanica* and *L. major* infections were statistically significant (p = 0.03), the rates of infections were at the same high level in both species (around 80% of analyzed females were positive on all examined days) and the development of location and stomodeal valve colonization ability were the same in both species without significant differences.

The ability of *L. major* to develop in the specific (selective) vector *P. papatasi* depends on the presence of species-specific modifications of the major surface glycoconjugate of promastigotes, lipophosphoglycan (LPG), which controls the parasite attachment to the sand fly gut [12,27]. The successful development of *L. turanica* in *P. papatasi* suggests that the LPG of this species must be identical or very similar to that of *L. major*.

Experiments on the development of *L. turanica* in two other sand flies distributed in the Middle East revealed that *L. turanica* develop late stage infections in *P.* (*Adlerius*) *arabicus* but not in *P.* (*Paraphlebotomus*) *sergenti*. In all *P. sergenti* examined, infection did not persist after digestion and defecation of the bloodmeal. This result is in agreement with previous findings that *P. arabicus* is a permissive vector susceptible to various *Leishmania* species, including *L. major*[23,28], while *P. sergenti* is the specific vector of *L. tropica* and is refractory to *L. major*[29,30]. Reports of *L. turanica* from other *Paraphlebotomus* species [1,9], however, suggest that vector competence for *Leishmania* may differ between members of this subgenus.

Using GFP and RFP-marked parasites we have experimentally demonstrated that *L. turanica* and *L. major* are able to develop in *P. papatasi* together, without any visible sign of competition. First, we used an initial infection dose of 1x10^7^ (resp. 5x10^6^*L. major* + 5x10^6^*L. turanica*) promastigotes/ml. Parasites did not differ in intensities or localization on all examined days, and the infections developed well with high rates of heavy infections for both species. Next, we decided to decrease the infection dose to better simulate natural conditions. Again, no significant differences were observed in the localization of infections and both *Leishmania* species colonized the stomodeal valve during mixed infections. Minor differences found in the infection intensities seem to be biologically unimportant, as other parameters, like colonization of the stomodeal valve, are more important for the vector status of the sand fly than the exact parasite number found in the midgut [23].

Despite the fact that both *Leishmania* species coexisted in the *P. papatasi* and *P. duboscqi* midgut for the relatively long period of two weeks (until the end of the experiment), the presence of dual fluorescing *L. major* / *L. turanica* hybrids was not demonstrated. Sadlova *et al.*[20] speculate that there may be species-specific differences among *Leishmania* species in their capacity for sexual reproduction and it should be noted that although *L. turanica* and *L. major* belong to the subgenus *Leishmania*, they are not closely related to each other [31]. Considering this, the similarity of LPG in *L. turanica* and *L. major* proposed here is likely to be a result of co-evolutionary convergence due to the adaptation of these two *Leishmania* species to the same sand fly vector.

## Competing interests

The authors declare that they have no competing interests.

## Authors’ contributions

ACH carried out the laboratory work of parasite development, drafted the first version of the manuscript and contributed to data analysis. JS performed data analysis of mixed infections. KK and VS carried out the detection of putative leishmania hybrids. JK performed the statistical analysis of data. MJ prepared GFP transfected *L. turanica*. ChS, SG and YM provided *L. turanica* strain. JV and PV designed the study, contributed to interpretation and finalized the manuscript. All authors read and approved the final version of the manuscript.
